# Chromosome-level assembly of the *Isodon lophanthoides* genome

**DOI:** 10.3389/fpls.2025.1528404

**Published:** 2025-03-05

**Authors:** Yubang Gao

**Affiliations:** College of Life Science, Nanyang Normal University, Nanyang, Henan, China

**Keywords:** genome, Chinese herbal medicine, *Isodon lophanthoides*, nanopore sequence, Hi-C assembly

## 1 Introduction

**Figure 1:**
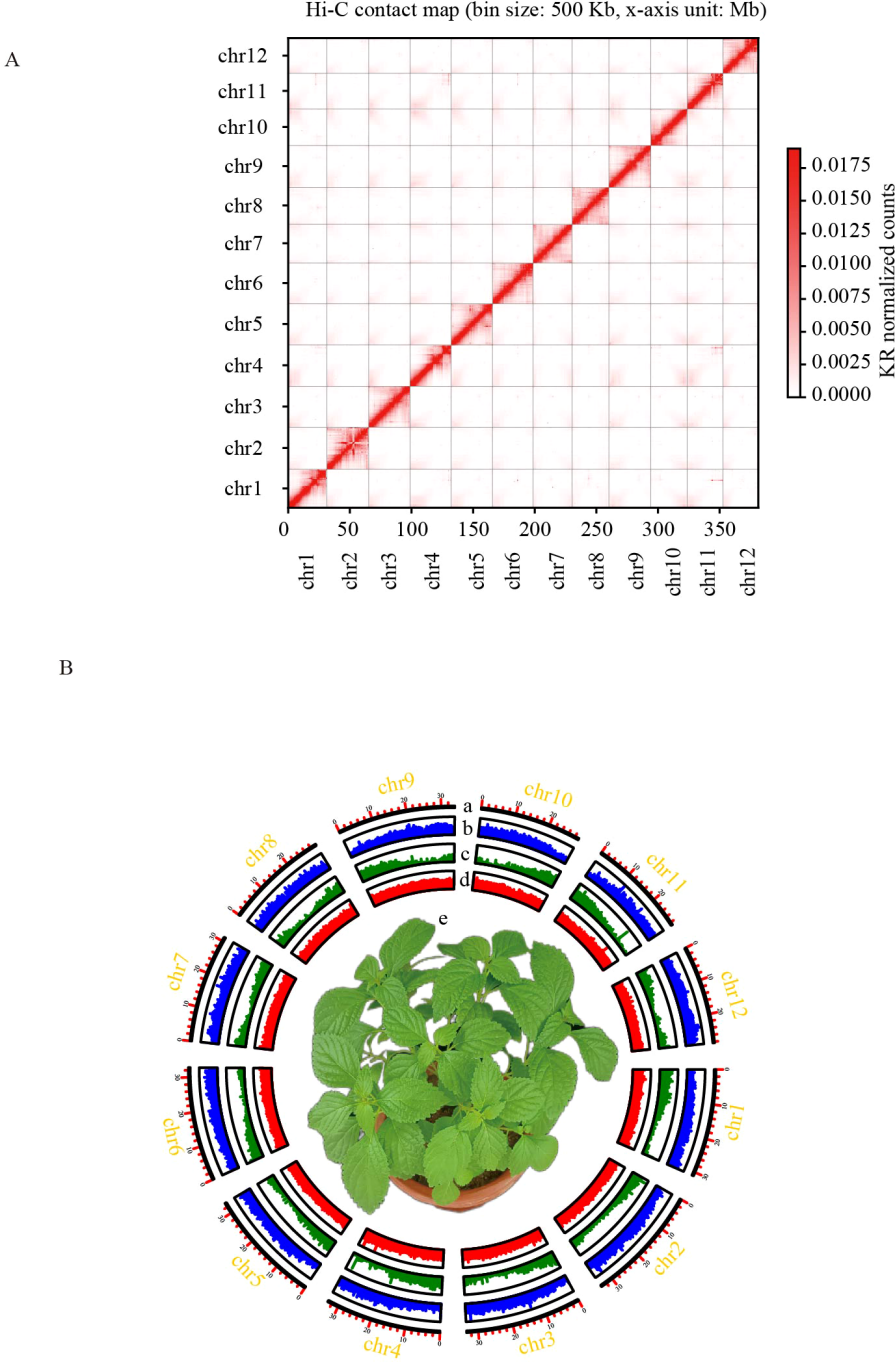
Chromosome-scale assembly of the *I. lophanthoides* genome. **(A)** Contact map of *I. lophanthoides* genome. **(B)** Circos plot displaying the 12 chromosomes in the *I. lophanthoides* genome. a. Length of each pseudochromosome (Mb). b. Distribution of repetitive sequences. c. Distribution of gene density. d. Distribution of the GC content. e. The phenotype of *I. lophanthoides* (The flower pot size was 15 cm).

*Isodon lophanthoides* (**Figure 1A**) is a perennial herb of the Lamiaceae family distributed across China, India, Myanmar, Nepal, and Vietnam (Wen et al., 2011; Zhang et al., 2022). *I. lophanthoides* contains a variety of bioactive compounds, such as terpenoids, flavonoids, phenolics, and polysaccharides (Lin et al., 2008; Wen et al., 2011; Zhou et al., 2014). *I. lophanthoides* is traditionally used to alleviate symptoms of acute jaundice hepatitis, arthritis, cholecystitis, enteritis, pharyngitis, ascariasis, and leprosy (Jiang et al., 2000). This herb is utilized in the preparation of therapeutic teas and instant granules. Additionally, it is used as an ingredient in soups and cooking. This plant plays a significant role in traditional Chinese medicine. It is cultivated extensively as a commercial raw material for the medicinal product “Xihuangcao”. The absence of genomic resources for *I. lophanthoides* has severely limited its genetic improvement and research on its active components. In this study, we assembled the first chromosome-level genome of *I. lophanthoides* and identified key genes involved in terpene biosynthesis. This work provides a valuable foundation for genetic improvement and exploring its active compounds’ biosynthetic pathways.

## 2 Materials and methods

### 2.1 Material collection and genome sequencing

Young leaves of *I. lophanthoides*, cultivated at the Artemisia Engineering Technology Center of Nanyang Normal University, were collected to extract high-quality DNA for genome sequencing. After DNA extraction, ultrasonic shearing was applied. The sequencing library was prepared through end-repair, adapter ligation, and amplification, followed by sequencing on the DNB-Seq T7 platform. For long reads, the sequencing library was prepared using the Oxford Nanopore ligation sequencing kit (SQK-LSK109). Sequencing was then performed on an R9 flow cell on the PromethION platform. For Hi-C reads, DNA was fixed in a 4% formaldehyde solution. Digestion was performed with the MboI enzyme, and digested fragments were labeled with biotin-14-dCTP. The crosslinked fragments were then blunt-end repaired and sequenced on the DNB-Seq T7 platform.

### 2.2 Genome survey

The k-mer method was used to estimate genome size and heterozygosity before genome assembly. The k-mer distribution was calculated from short reads using Jellyfish (Marcais and Kingsford, 2012) with k-mer length set to 21. The genome size and heterozygosity rate was estimated using the GenomeScope2 (Ranallo-Benavidez et al., 2020).

### 2.3 Genome assembly and gene annotation

Genome assembly was conducted using NextDenovo (Hu et al., 2024) with the overlap-layout-consensus algorithm and default parameters. NextPolish (Hu et al., 2020) was used to polish the genome assembly, applying two rounds of long-read and four rounds of short-read data correction. Hi-C reads were aligned to contigs using Juicer (Durand et al., 2016) and BWA (Jung and Han, 2022), after which the 3D-DNA pipeline (Dudchenko et al., 2017) corrected misassemblies and ordered contigs, integrating them into scaffolds. Manual inspection of scaffolds was then performed using Juicebox Assembly Tools. The final chromosome-length scaffolds were constructed using the 3D-DNA pipeline, with all computational tools run using default parameters. Misassemblies were identified and corrected based on irregular contact patterns in Hi-C data.

Repeat elements in genomes were identified using RepeatModeler (Flynn et al., 2020), and the repeat library was then processed with RepeatMasker (Tarailo-Graovac and Chen, 2009) to annotate repeats across the genome. Transposable elements (TEs) were classified using TEsorter (Zhang et al., 2022). Simple sequence repeat (SSR) markers were predicted using MISA (Beier et al., 2017). Protein-coding genes in the *I. lophanthoides* genome were identified using an integrative strategy that combined ab initio prediction, protein homology searches, and RNA sequencing data. For ab initio prediction, we used Augustus (Stanke et al., 2006), SNAP (Korf, 2004), GlimmerHMM (Majoros et al., 2004), and GeneMark-ET (Brůna et al., 2020) to identify gene structures in the repeat-masked genome. For protein homology prediction, protein data from sequenced Lamiaceae species were downloaded from the NCBI database and aligned for homology assessment. Additionally, HISAT2 (Kim et al., 2019) was used to map RNA-seq data (PRJNA679679) from various tissues to the genome. PASA was used to predict open reading frames. EVidenceModeler (Haas et al., 2008) integrated results from the three methods, enabling a unified gene prediction. Functional annotation was performed using BLAST (Ye et al., 2006) against NR, SwissProt, eggNOG, InterPro, GO, and KEGG databases. Functional annotations for protein-coding genes were integrated using the above methods.

### 2.4 Phylogenetic analysis

Protein sequences of *A. trichopoda*, *O. sativa*, *V. vinifera*, *T. cacao*, *A. thaliana*, *S. lycopersicum*, *C. canephora*, *T. grandis*, *L. japonicus*, *S. miltiorrhiza*, *I. rubescens*, and *A. decumbens* were downloaded for subsequent analyses. OrthoVenn3 (Sun et al., 2023) was used for orthology, phylogenetic, and gene family analyses. Pairwise sequence similarity was determined using BLASTP and OrthoMCL (Li et al., 2003) Markov clustering. Phylogenetic trees were constructed using FastTree2 (Price et al., 2010) with the maximum likelihood method and the JTT+CAT model, with node reliability assessed by the SH test. A divergence tree was constructed using single-copy genes and fossil evidence. Divergence times between *A. thaliana* and *T. cacao*, *S. lycopersicum* and *C. canephora*, *A. thaliana* and *V. vinifera*, *A. trichopoda* and *V. vinifera*, and *L. japonicus* and *T. grandis* were estimated using r8s (Sanderson, 2003). CAFE (Mendes et al., 2020) was used to compare cluster size differences between ancestors and each species to determine gene family expansions and contractions. A random birth-and-death model was applied to assess gene family changes across lineages in the phylogenetic tree. Conditional likelihood was used as the test statistic, with p-values of ≤ 0.01 considered significant.

### 2.5 Duplicated gene analysis

*I. lophanthoides* protein sequences were compared to identify homologous blocks. The MCScanX (Wang et al., 2012) pipeline was applied with default settings to map homologous blocks within species. The YN model in KaKs_Calculator 2.0 (Wang et al., 2010) was used to calculate nonsynonymous (Ka) and synonymous (Ks) substitution rates, as well as their ratio (Ka/Ks), for duplicate gene pairs.

## 3 Data

### 3.1 Genome assembly

DNA was isolated from *I. lophanthoides* samples cultivated in the laboratory. Genome size and heterozygosity were estimated using DNB short-read sequencing data. The estimated genome size from short reads was 365,686,342 bp, with a heterozygosity rate of 0.64% (k-mer length = 21). DNA from the same plant was used to assemble the *I. lophanthoides* genome with a combination of Nanopore and Hi-C technologies (**Supplementary Table S1**). Assembly with Nanopore long reads produced a genome with a total length of 379,974,750 bp, containing 70 contigs (N50 = 17,265,197 bp). After Hi-C scaffolding, 378,710,417 bp (99.67%) of the sequence was placed into 12 linkage groups (**Figure 1A**). These linkage groups corresponded to the 12 chromosomes of *I. lophanthoides* (N50 = 32,786,395 bp). BUSCO assessment showed that the assembly covered 98% of the single-copy orthologs in the embryophyta_odb10 database (1,614 genes; **Supplementary Table S2**). The consensus quality value (QV) was 35.77, indicating that the genome is highly accurate. The genome’s LAI value is 13.78, reaching the level of the reference genome.

### 3.2 Gene prediction and gene annotation

50.52% of the genome assembly consisted of repetitive elements, with half of this proportion (30% of the genome) being retrotransposons. This retrotransposon content is similar to that in *I. rubescens*. In the *I. lophanthoides* genome, 9.38% of the copies were identified as Copia elements, and 9.93% as Gypsy elements. We further classified transposable elements (TEs) using Tesort (Zhang et al., 2022), identifying 5,880 Helitrons, 4,015 LINEs, 94,428 LTRs, and 13,042 TIRs. Additionally, 153,599 SSR markers were predicted using MISA (Beier et al., 2017).

EVidenceModeler was used to integrate outputs from transcriptome data, ab initio predictions, and homology-based predictions. A total of 30,641 genes were identified, of which 28,541 were protein-coding (**Figure 1B**). These genes contained an average of 4.8 exons, with an average coding sequence (CDS) length of 1,112 bp (**Supplementary Table S3**). Functional annotation of 26,492 protein-coding genes (92.8%) was achieved using GO, NR, KEGG, TAIR, and InterProScan databases. A total of 40 genes were associated with terpene metabolism, including 12 genes in the MEA pathway and 28 in the MEP pathway (**Supplementary Table S4**). Non-coding RNA prediction identified 297 rRNAs, 541 tRNAs, 101 miRNAs, and 341 snRNAs.

### 3.3 Comparative genomic analysis of *I. lophanthoides* with other plants

**Figure 2:**
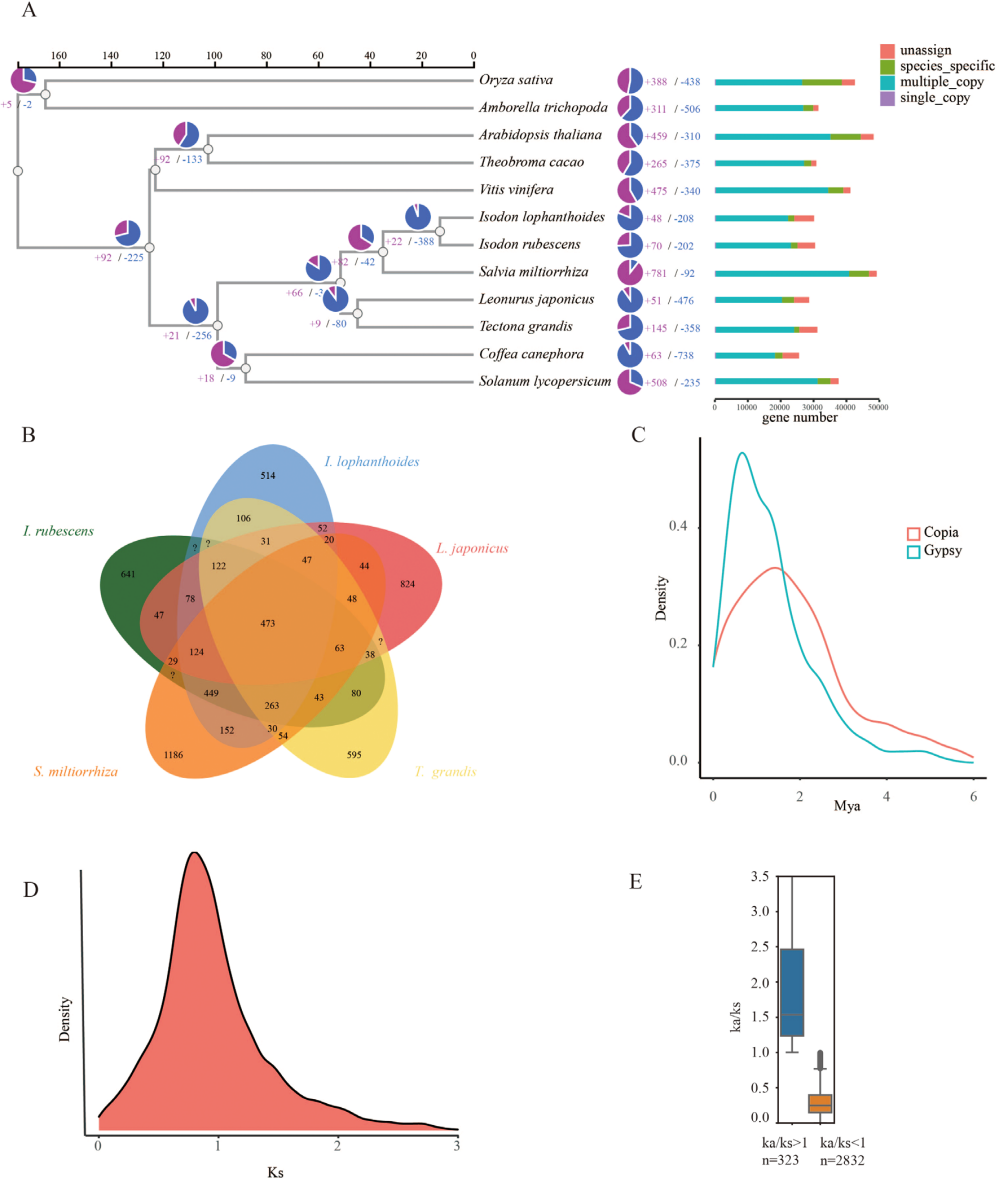
Evolutionary analysis of the *I. lophanthoides* genome. **(A)** A phylogenetic tree based on shared single-copy gene families, gene family expansions, and contractions among *I. lophanthoides* and ten other species. The bar chart on the right displays gene family clustering in *I. lophanthoides* and ten other plant species. **(B)** Venn Diagram Representation of Gene Family Overlaps and Specificities Among *I. lophanthoides*, *I. rubescens L. japonicus*, *T. grandis*, and *S. miltiorrhiza* in Labiatae. **(C)** Density plot showing the burst of LTR-RTs in *I. lophanthoides*. **(D)** Ks value distribution plot for orthologous gene sets of *I. lophanthoides*. **(E)** Ka/Ks value distribution plot for orthologous gene sets of *I. lophanthoides*.

To determine the evolutionary relationships between *I. lophanthoides*, *I. rubescens*, and other plant species, a phylogenetic tree was constructed using a total of 427,238 proteins from 12 plant species (**Supplementary Table S5**). These proteins were clustered into 35,165 orthogroups, of which 282 were single-copy genes (**Supplementary Table S6**). With known divergence times added, the phylogenetic tree indicated that the common ancestor of *I. lophanthoides* and *I. rubescens* diverged approximately 12.988 million years ago (MYA) (**Figure 2A**). In *I. lophanthoides*, 48 gene families showed significant expansion and 208 showed significant contraction. The number of expanded gene families was smaller than in *I. rubescens*. Compared with other Lamiaceae species, *I. lophanthoides* had the fewest unique gene families (**Figure 2B**). A transposon burst occurred in *I. rubescens* gene families around 1 MYA (**Figure 2C**). The Ks method was used to analyze orthologous gene pairs, revealing no lineage-specific whole-genome duplication events other than the shared peak in Lamiaceae (**Figure 2D**). Further analysis of selection-affected genes identified 323 genes under positive selection and 2,832 under negative selection (**Figure 2E**). Genes under positive selection were enriched in processes such as “response to salicylic acid” (**Supplementary Figure S3**).

## Data Availability

The datasets presented in this study can be found in online repositories. The names of the repository/repositories and accession number(s) can be found below: https://www.ncbi.nlm.nih.gov/, SRR29855129, SRR28822717, SRR29849713 https://figshare.com/, https://figshare.com/s/791b7bef4735829aaf3e.
