# Supplementary material for: Chromosome-level assembly of the Isodon lophanthoides genome
Source: Front Plant Sci. 2025 Mar 5;16:1528404. doi: 10.3389/fpls.2025.1528404 (PMC11919858; doi:10.3389/fpls.2025.1528404)
Supplement: Supplementary file 1 [file DataSheet1.docx]

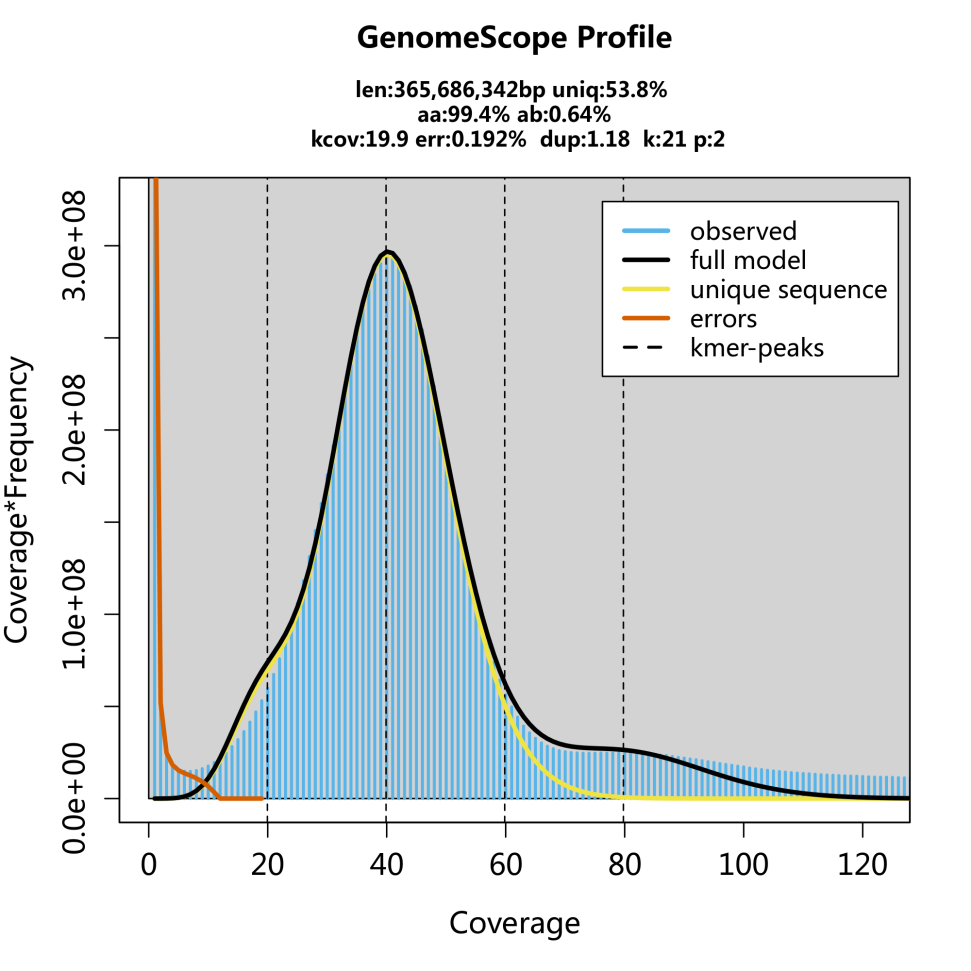


Fig. S1 Genome survey of *I. lophanthoides*.


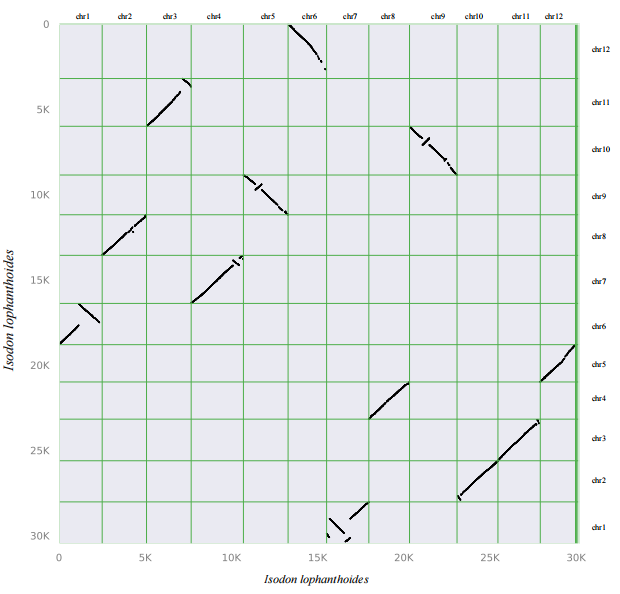


Fig. S2 Colinearity analysis of *I. lophanthoides* and *I. rubescens*.


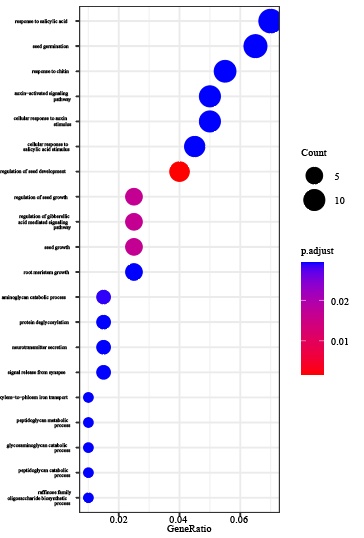


Fig. S3 GO enrichment Analysis of positively selected genes.
